# Gut bacteriome dynamics in high altitude-adapted chicken lines: a key to future poultry therapeutics

**DOI:** 10.1038/s41598-025-96178-1

**Published:** 2025-04-07

**Authors:** Neha R. Bhagat, Vijay K. Bharti, Geeta Shukla, Praveen Rishi, O. P. Chaurasia

**Affiliations:** 1 DRDO-Defence Institute of High Altitude Research (DIHAR), Leh, UT Ladakh 194101 India; 2 Department of Microbiology, Panjab University, Chandigarh, India

**Keywords:** Adaptation, DIHAR-chicken, Gut-bacteriome, High-altitude, Microbial diversity, Ecology, Microbiology, Zoology, Environmental sciences

## Abstract

High-altitude-adapted chickens harbor a unique gut bacteriome essential for their survival under extremely cold and hypoxic environment, however, little is known about their population and functional dynamics, limiting their application in poultry production. Hence, this study employed amplicon-based metagenomics to examine the gut bacterial diversity and their functional profile in two high-altitude-adapted chicken lines, e.g. LEHBRO-1 and LEHBRO-3. The results revealed significant variations in taxonomic abundance at the phylum level, with Firmicutes, Proteobacteria, Bacteroidetes, and Actinobacteria predominating in LEHBRO-1, whereas Firmicutes, Proteobacteria, Bacteroidetes, Planctomycetes, and Actinobacteria predominated in LEHBRO-3. Genus-level diversity and Linear Discriminant Analysis Effect Size (LEfSe) biomarker analysis also substantiated the differences in the gut bacterial communities between the two chicken lines. Furthermore, functional profiling revealed enrichment of carbohydrate, nucleotide, lipid, amino acid, fatty acid, energy, and glycan metabolic pathways in the gut bacteriomes of these high-altitude chicken lines. The Statistical Analysis of Metagenomic Profiles (STAMP) for metabolic profiling identified a significant difference in purine and protein metabolism between these two chicken lines. These findings indicate the unique gut bacteriome and their functional diversity in high-altitude-adapted chickens, which would provide a foundation for future research on gut therapeutics to improve chicken health and productivity in high-altitude areas.

**Supplementary Information:**

The online version contains supplementary material available at 10.1038/s41598-025-96178-1.

## Introduction

At high altitudes, hypoxia, and cold stress present substantial challenges for low-lander chickens such as ascites and reduced performance, resulting in adverse effects on their survival and productivity^1^. On the other hand, high-altitude-adapted chickens, such as the LEHBRO-1 and LEHBRO-3 lines developed by the Defense Institute of High-Altitude Research (DIHAR) in Leh-Ladakh, exhibit unique physiological and genetic traits that enhance their ability to thrive in these challenging terrains. These adapted chicken lines play a crucial role in producing meat and eggs, an essential source of nutrition (protein and essential nutrients) for the local communities in these regions. Moreover, the increasing demand for poultry products highlights the urgent need to address food and nutritional security issues in the high-altitude region^1,2^. Therefore, researchers are exploring feed additives and supplements for improving adaptation and productivity in chickens at high altitudes and other similar parts of the country^2–4^. While these strategies show promise, their effectiveness has been inconsistent at high altitudes, limiting their application in poultry production^2,3^. This inconsistency is primarily attributed to the difference in the gut microbiome of high-altitude chickens than the low-altitude chickens^1,2^.

Recently, the gut microbiota of chickens has gained enormous interest for its role in growth, health, nutrient utilization, metabolism, immunity, production performance, energy regulation and region-specific adaptation to different climatic conditions due to their secreted metabolites^6^. These gut microbes are well known for modulating the host health, and production performance, particularly in extreme conditions like hypoxia and cold stress^5,7,8^. Hence, it is hypothesized that high-altitude-adapted chickens may rely on unique gut bacteria that produce secretory metabolites to support their survival and performance at high altitudes compared to other chickens^8–12^. Moreover, this microbial diversity is believed to differ across chicken lines/strain/breed, enabling them to tolerate the harsh climates of high-altitude regions^8,9^. However, the lack of knowledge of the gut-microbes, and their functional dynamics in different breeds of high-altitude adapted chickens at Leh-Ladakh hampers the development of effective feed additives tailored to these conditions^2^. In addition, there is a lack of comprehensive studies characterizing the beneficial microbial diversity, functional dynamics, and specific metabolite-synthesizing pathways in different lines of high-altitude-adapted chickens, especially in Leh-Ladakh.

To address this knowledge gap, 16 S rRNA amplicon-based metagenomics offers a cost-effective, powerful and versatile tool for studying culturable as well as non-culturable bacterial diversity, providing insights into their population and next-generation probiotics dynamics without the need to culture individual species^13,14^. Among bioinformatics platforms, EzBioCloud stands out for its reliability and ease of use in taxonomic classification, surpassing alternatives like SILVA, RDP, GTDB, and Greengenes^15–17^. Hence, the present study investigated the gut bacterial diversity and functional profile of two different high-altitude adapted chicken lines, i.e., the DIHAR Red line (LEHBRO-1) and DIHAR Black line (LEHBRO-3) using 16 S V3‒V4 amplicon sequencing followed by EzBioCloud MTP analysis-based bioinformatic analysis to determine the gut bacteriome dynamics in high-altitude-adapted chicken lines.

This study has brought new knowledge on beneficial gut bacterial diversity, and their important metabolic pathways in high altitude-adapted lines of chickens. Consequently, the current study provides distinctive findings, which will be valuable in developing future therapeutics with bacteria having probiotic or industrially relevant-metabolite-producing capabilities, and will ultimately have beneficial implications on poultry production and the probiotics industry.

## Materials and methods

### Experimental chicken and their housing conditions

The present study was carried out on fecal samples collected from two lines of high-altitude-adapted chickens (*Gallus gallus domesticus*), i.e., the DIHAR-Red Line (LEHBRO-1) and DIHAR-Black Line (LEHBRO-3). The DIHAR-Red Line (LEHBRO-1) was developed from Red Cornish, while the DIHAR-Black Line (LEHBRO-3) was developed from the Black Rock breed at Leh-Ladakh with a feed conversion ratio of 4–5. These DIHAR chicken lines are well adapted to high altitude conditions and have better resilience, growth, and reproductive performance. These high-altitude-adapted chicken lines are reared at the Institute Experimental Facility located at an altitude of 3,500 m, i.e., Leh (34.152588 N, 77.577049E), Ladakh, India, under a deep-litter system based solar poultry housing. This facility provides a 16-hour light period, maintains an ambient temperature of 25–27 °C, and is equipped with a heating system, which overcomes the extreme cold temperature prevalent in high-altitude, Leh-Ladakh. All these chickens were fed with commercially available readymade feed as per the standard ration scale with free access to water and no antibiotics or drugs were given in their feed during rearing, and the experimental period. All the experimental protocols were approved by the DIHAR institutional animal ethics committee (IAEC), and methods were carried out in accordance with the guidelines and regulations of CCSEA, India, and ARRIVE.

### Sample collection

Since the fecal microbial community mirrors the microbial diversity of the gastrointestinal tract of animals, a total of 08 freshly defecated fecal samples were collected from healthy adult laying chicken lines (39–41 weeks of age), LEHBRO-1 (*n* = 4) and LEHBRO-3 (*n* = 4) in a labeled sterile centrifuge tube^18^ and further processed for targeted 16 S (V3-V4) amplicon metagenomic analysis.

### DNA extraction, library preparation, and sequencing

In brief, Prokaryotic genomic DNA was extracted from each sample following a standard protocol and quantified using a Nanodrop1000 spectrophotometer. Further, the extracted DNA was prepared for sequencing through library preparation as per the Illumina 16 S amplicon-sequencing library protocol to amplify the V3 and V4 regions, which involved 4 steps, i.e., (1) fragmentation, (2) ligation of adapter sequences, (3) insert-size selection with the TapeStation 4150 system (Agilent Technologies) employing highly sensitive D1000 screen tapes (Agilent #5067–5582), and (4) library quantification, which was executed with a Qubit 4.0 fluorometer (Thermo Fisher Scientific #Q33238) equipped with a DNA HS assay kit (Thermo Fisher Scientific #Q32851). These prepared libraries were then sequenced on the Novaseq 6000 platform (Illumina, San Diego, CA, USA) using standard kits and protocol to yield 250 × 2 paired-end reads. For this, Solution Molsys Pvt Ltd., Banglore, helped as an outsourcing agency.

### Analysis of sequencing data

The paired-end reads generated from the NovaSeq 6000 platform were self-analyzed for bioinformatic analysis and comparative studies between the two lines of chickens via upload to the EzBioCloud 16 S rRNA gene-based Microbiome Taxonomic Profiling (MTP) server (https://www.ezbiocloud.net/contents/16smtp) (ChunLab, Inc., Seoul, Republic of Korea)^16,17,19,20^. In brief, the Illumina paired-end reads were subjected to MTP taxonomic profile analysis via the PKSSU4 pipeline and analyzed for the 16 S bacterial V3-V4 region. This software first pre-processed the reads by trimming primers used during PCR, then filtered the low-quality reads (Q < 20) and subsequently, the quality reads were processed for denoising and removal of chimeric sequences for generating valid reads. After that, the uploaded MTP Data sets were selected for comparative microbiome profiling and the valid reads were screened by the software for OTU picking via EzBioCloud-Open reference UCLUST-MC2 at a 97% similarity cutoff threshold for taxonomic analysis, Firmicutes: Bacteroidetes ratio, alpha diversity (abundance-based coverage estimator (ACE), Chao1, Shannon, Simpson, Jackniff, and phylogenetic diversity), beta diversity (Jensen, Unifrac, Bray Curtis and generalized UniFrac), and taxonomic biomarkers (Lefse) among the lines of chickens^17,20^. On the basis of these findings, bacteria exhibiting beneficial attributes as probiotics or producers of industrially relevant secondary metabolites were subsequently identified and further reviewed for their functional capabilities on the basis of published reported scientific articles and later through other functional profiling databases.

### Functional profile of gut bacterial communities

To identify the differentially abundant profile of microbial pathways across the two cohorts, predictive functional profiling was performed via the EzBioCloud-based PICRUSt and the Phylogenetic Investigation of Communities by Reconstruction of Unobserved States 2 (PICRUSt2)^21^ database. EzBioCloud software is known to provide functional biomarkers analysis using the PICRUST pathway, and therefore, it was used to compare the differences in functional biomarkers between the chicken lines^22^. In addition, Picrust2 is the best-known database widely used for functional analysis to predict metabolic pathways based on taxonomic data with a lesser number of false positive rates than other software, i.e., Piphillin and Tax4fun2^23^. After that, the results were self-analyzed, and the pathways with a mean frequency of more than 0.7% were considered enriched pathways in the studied chicken lines.

### Statistical analysis

In EzBioCloud, the statistical significance of the results was demonstrated using the Wilcoxon rank-sum test to compare the α diversity indices, and permutational multivariate analysis of variance (PERMANOVA) was measured to compare the beta diversity between chicken lines, both at the *p* < 0.05 significance level. Further, LEfSe was performed to determine statistically significant enrichment in the assigned taxonomic profiles between the two chicken lines in terms of the magnitude of the change in an OTU versus the p-value of the OTUs through EzBioCloud software. Additionally, the Lefse (LDA effect > 2; *p* < 0.05) and Kruskal Wallis H test (*p* < 0.05) were used to analyze the EzBioCloud (PICRUSt) data, and the Statistical Analysis of Metagenomic Profiles (STAMP) (*p* < 0.05) to analyze the PICRUST2 data to identify the statistical differences of functional profile in gut bacteriome of the two high-altitude-adapted chickens.

### Merit and limitations of the study

This is the first investigation aimed at exploring and identifying the potential bacterial communities in high-altitude-adapted chickens and their functional pathways in extreme climatic environments (Leh-Ladakh). Although the sample size (*n* = 4) selected for each line of chickens was relatively small in the present study, however, size is sufficient as per the objectives of the present study and meets the statistical significance of the study. This sample size was our best attempt, considering the financial viability of the study.

## Results

### Sequencing data

After quality processing, on average, 3799.5 ± 227.03 OTU counts per sample were obtained, with a median number of reads as 3872 OTU counts per sample and an average sequence length of 421.5 ± 3.10 bp from all the fecal samples, as shown in a supplementary Table 1. More than 99% good coverage quality was observed in samples of both chicken lines, i.e., 99.82 ± 0.04 for LEHBRO-1 chickens and 99.86 ± 0.03 for LEHBRO-3 chickens.

### Taxonomy

**Fig. 1:**
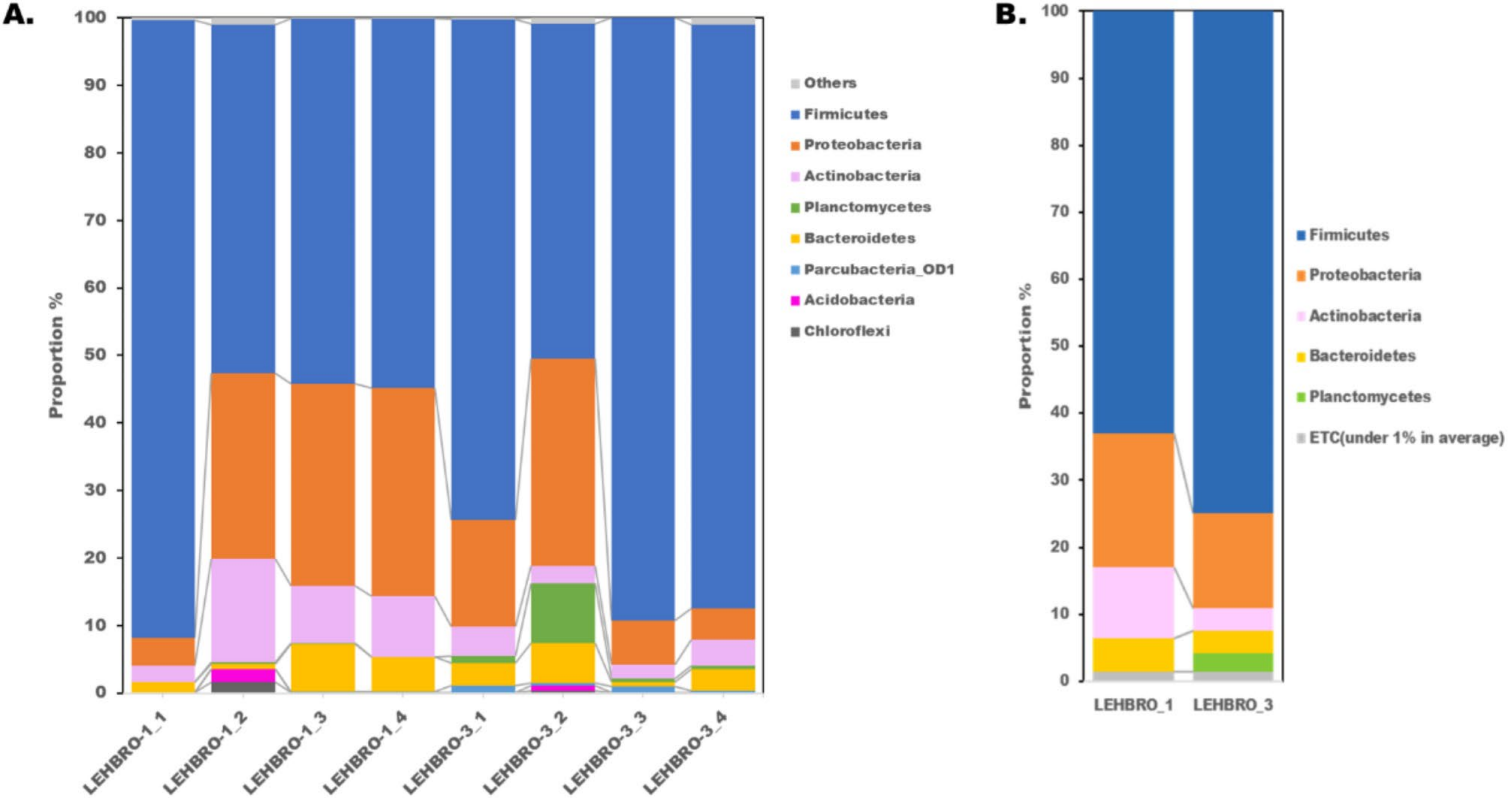
Comparative analysis of Gut Bacterial Diversity at the Phylum Level in High-Altitude-Adapted Chicken Lines, (**A**) List of abundant phyla present in each biological replicate from high-altitude-adapted chicken lines (relative abundance > 1%); (**B**) Overall relative abundance of phyla in high-altitude-adapted chicken lines (relative mean abundance: cutoff > 1%).

Comparative gut bacterial composition analysis disclosed a marked variation in the relative abundance of taxa between the two lines of chickens (Fig. 1). At the phylum level, all sequences were classified into only four phyla (> 1% in average), i.e., Firmicutes (62.97%), Proteobacteria (20.05%), Actinobacteria (10.58%) and Bacteroidetes (4.93%), in the high-altitude-adapted LEHBRO-1 chicken line, whereas five phyla, i.e., Firmicutes (74.85%), Proteobacteria (14.26%), Actinobacteria (3.31%), Bacteroidetes (3.42%) and Planctomycetes (2.69%), were identified in the LEHBRO-3 chicken line, as shown in Fig. 1.

**Fig. 2:**
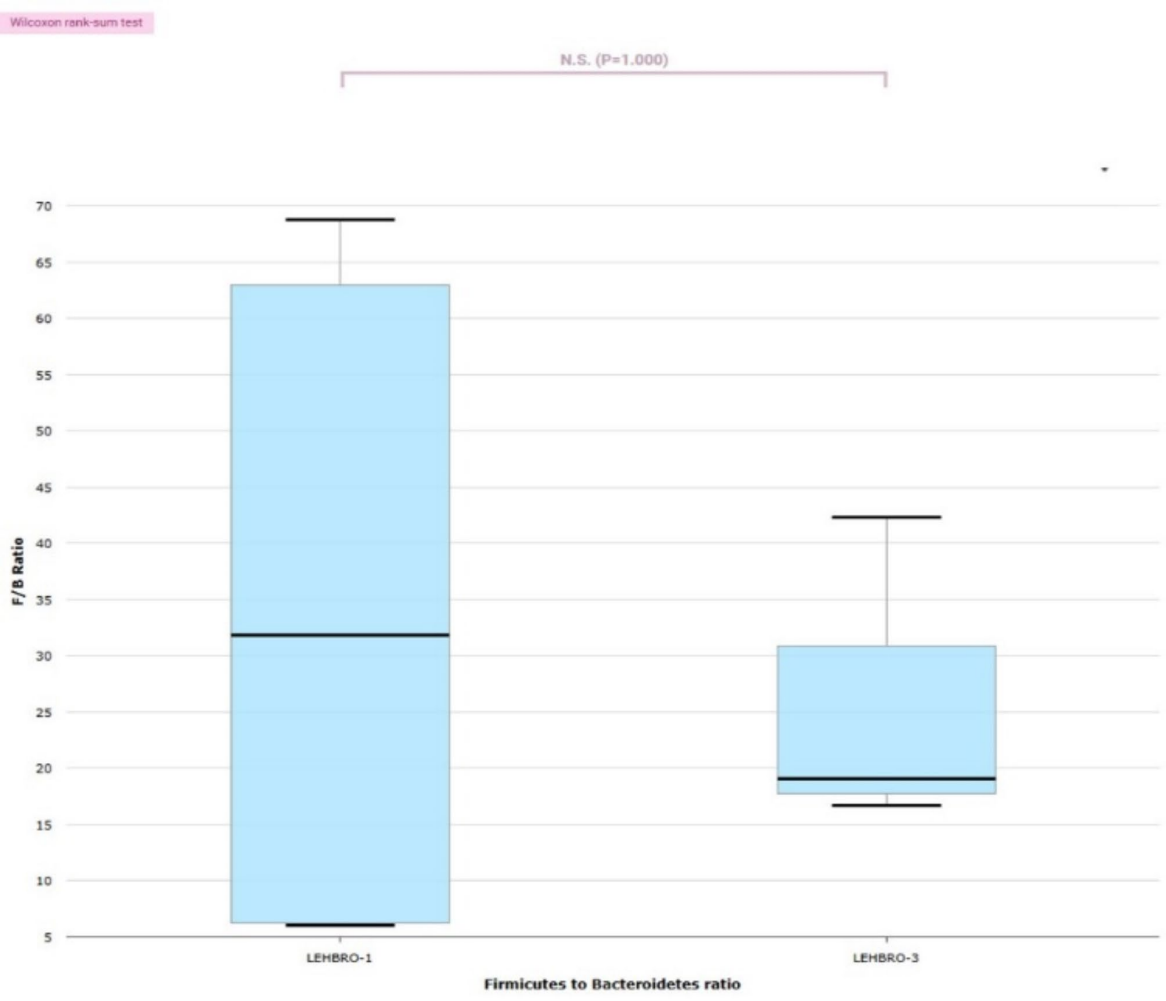
Comparison of the Firmicutes-to-Bacteroidetes ratio (F:B ratio) in two different lines of high-altitude-adapted chickens.

Moreover, the Firmicutes-to-Bacteroidetes ratio in LEHBRO-1 chickens was LEHBRO-3 chickens, as shown in Fig. 2 and data at family, class and order levels are presented in supplementary Figs. 1, 2 and 3.

**Fig. 3:**
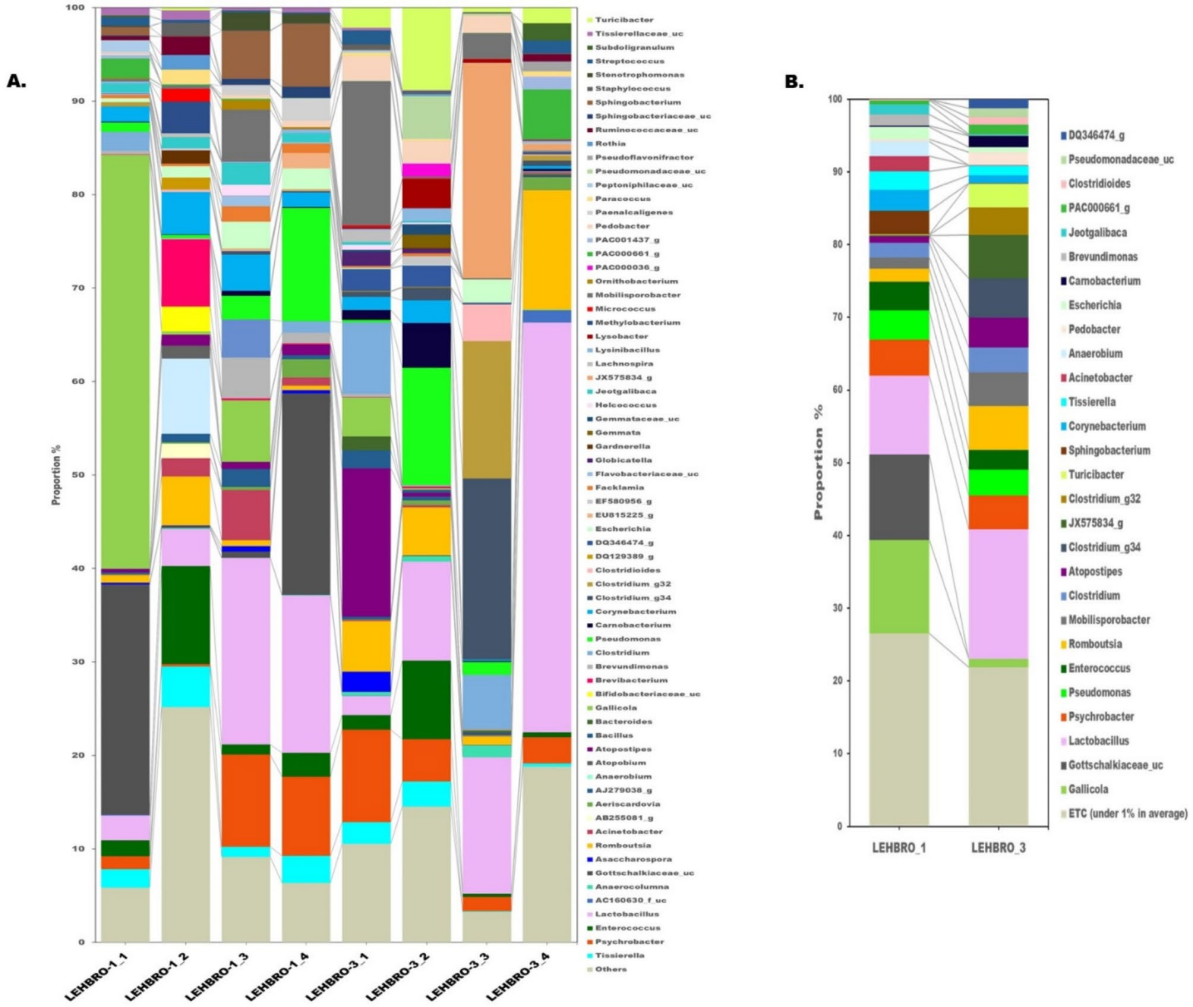
Comparative analysis of Gut Bacterial Diversity at the Genus Level in High-Altitude-Adapted Chicken Lines (> 1%), (**A**) List of abundant genera present in each sample from high-altitude-adapted chicken lines (relative abundance: cutoff > 1%); (**B**) Overall relative abundance of the genus in high-altitude-adapted chicken lines (relative mean abundance: cutoff > 1%).

At the genus level, a comparative analysis between the two chicken lines is presented in Fig. 3. On average, a total number of 487 genera of bacteria were identified in the LEHBRO-1 chicken line, and 534 genera were identified in the LEHBRO-3 chicken line. Among these identified genera, those with an average frequency greater than 1% were considered relatively abundant in high-altitude-adapted chickens. Consequently, a total of 18 bacterial genera (> 1% in average), i.e., *Gallicola* (12.81%), *Gottschalkiaceae*_uc (11.77%), *Lactobacillus* (10.83%), *Psychrobacter* (4.99%), *Pseudomonas* (4%), *Enterococcus* (3.97%), *Sphingobacterium* (3.19%), *Corynebacterium* (2.9%), *Tissierella* (2.57%), *Acinetobacter* (2.06%), *Anaerobium* (2.02%), *Clostridium* (1.98%), *Brevibacterium* (1.87%), *Romboutsia* (1.8%), *Escherichia* (1.7%), *Mobilisporobacter* (1.54%), *Brevundimonas* (1.51%), and *Jeotgalibaca* (1.4%), were relatively abundant in the LEHBRO-1 chicken line. On the contrary, a total of 21 bacterial genera (> 1% in average), i.e., *Lactobacillus* (17.76%), *Romboutsia* (6.06%), JX575834_g (5.93%), *Clostridium*_g34 (5.46%), *Psychrobacter* (4.69%), *Mobilisporobacter* (4.62%), *Atopostipes* (4.08%), *Clostridium*_g32 (3.8%), *Pseudomonas* (3.55%), Clostridium (3.42%), *Turicibacter* (3.27%), *Enterococcus* (2.71%), *Pedobacter* (1.77%), *Carnobacterium* (1.55%), *Tissierella* (1.35%), PAC000661_g (1.34%), DQ346474_g (1.26%), *Pseudomonadaceae*_uc (1.19%), *Gallicola* (1.12%), *Corynebacterium* (1.07%) and *Clostridioides* (1.01%), were relatively abundant in LEHBRO-3 chickens. In both chicken lines, the relatively abundant genera *Clostridium*,* Corynebacterium*,* Enterococcus*,* Gallicola*,* Lactobacillus*,* Mobilisporobacter*,* Pseudomonas*,* Psychrobacter*,* Romboutsia*, and *Tissierella* were commonly found, however, the presence of other genera were chicken line-specific in nature.

**Fig. 4:**
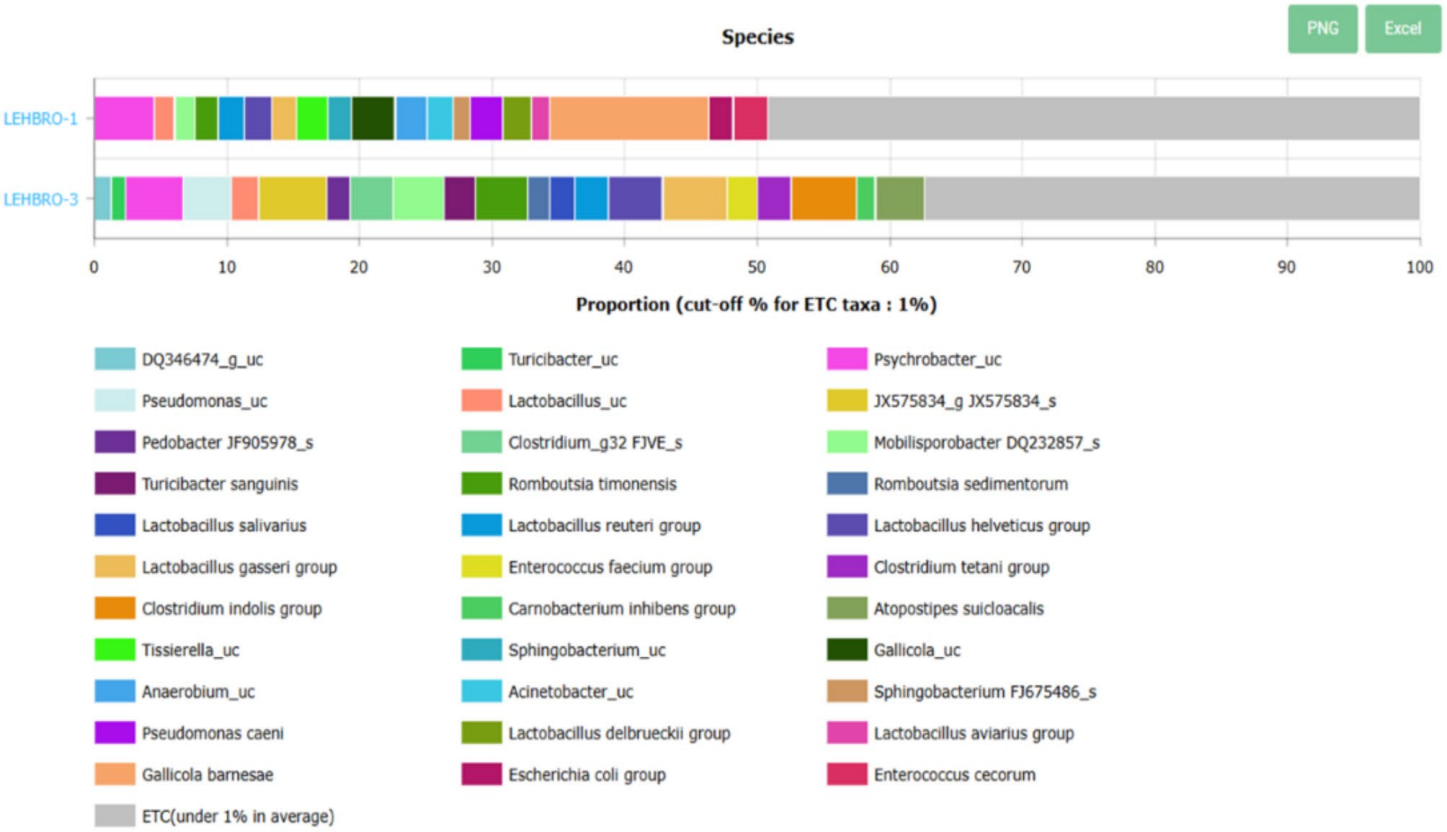
Comparative relative abundance of gut microbes at the species level in two different lines of high-altitude-adapted chickens (cutoff > 1%).

While 16 S amplicon sequencing offers reliable data up to the genus level only, however, it can provide a predictive profile of microbes at the species level. In addition, EzBioCloud MTP, in comparison to other databases, can potentially provide a probable bacterial profile at the species level (> 97% cutoff), which is useful for identifying unculturable bacterial species, as well as some culturable bacterial species, as demonstrated in Fig. 4.

### LefSe

**Fig. 5:**
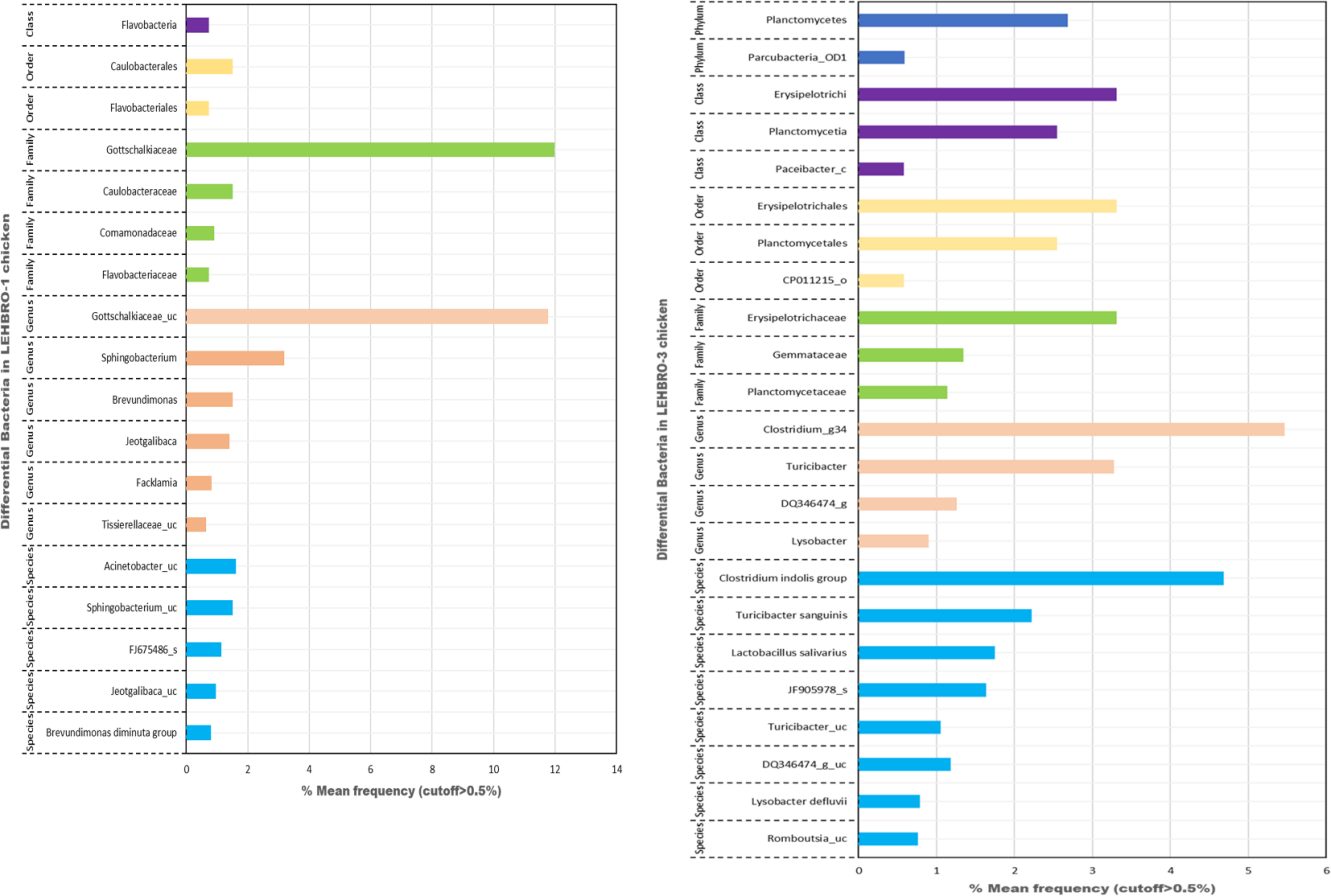
LEfSe analysis of the gut bacteriome across different taxonomic levels in high-altitude-adapted chicken lines (cutoff > 0.5% mean frequency).

In addition to relative abundance at the taxonomic level, linear discriminant analysis effect size (LEfSe) was also studied to identify specific taxa that are enriched in the gut microbiome of each chicken line. The results revealed that a total of 2 phyla, 4 classes, 5 orders, 7 families, and 10 genera were distinctive among the two lines of chickens, with a relative frequency of more than 0.5. The phyla planctomycetes and Parcubacteria_OD1 were different and highly present in LEHBRO-3 chickens. Further, the taxonomic category data revealed that individually, a total of 1 class, 2 orders, 4 families, and 6 genera were distinctive among the LEHBRO-1 chicken lines, whereas 2 phyla, 3 classes, 3 orders, 3 families, and 4 genera of the gut bacteriome were distinctive for the LEHBRO-3 chicken line, as shown in Fig. 5.

### Alpha (α)-diversity

**Fig. 6:**
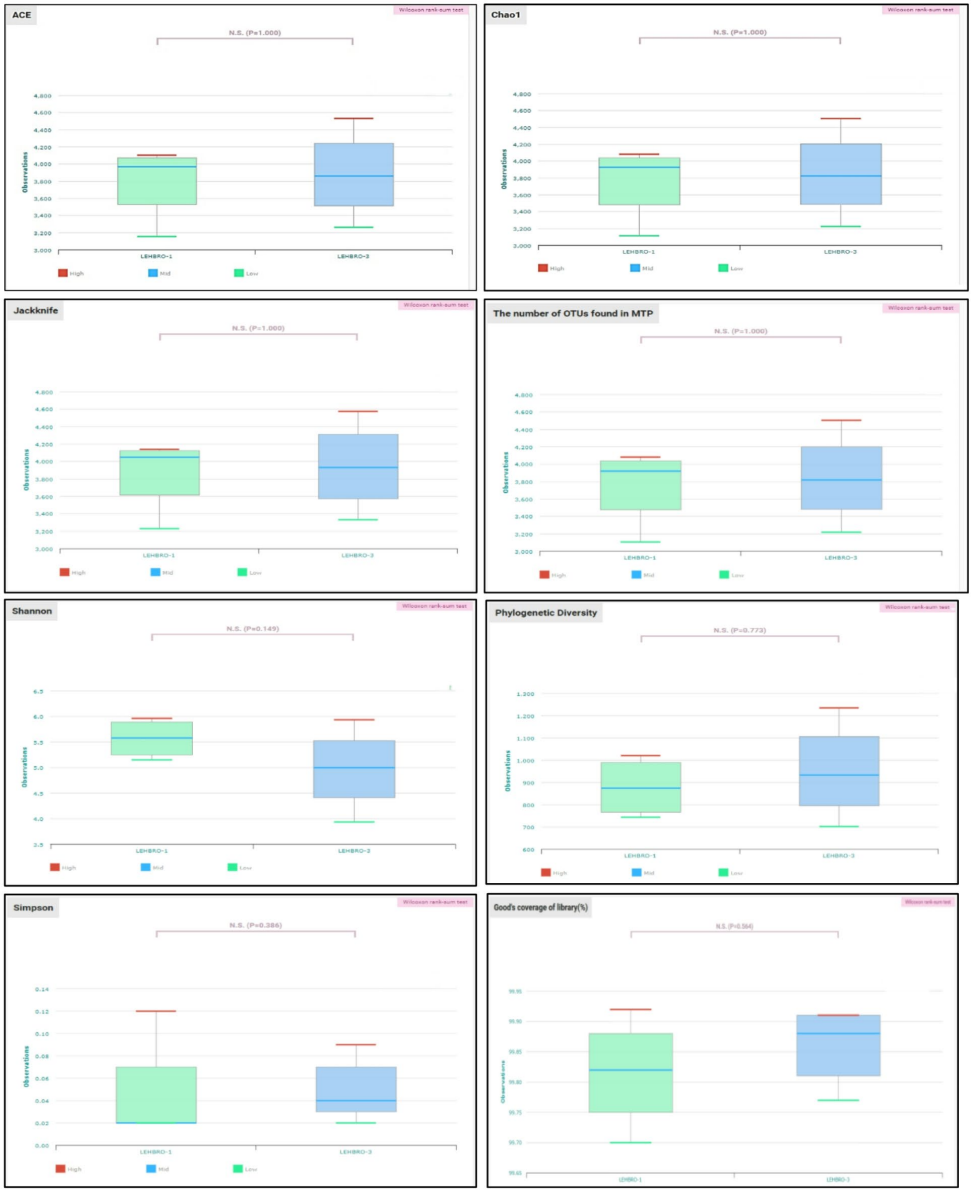
Alpha (α)-diversity indices of the gut bacteriome in two high-altitude-adapted chicken lines.

Alpha diversity provides a structural ecological community profile in terms of evenness (distribution of abundances among the groups), richness (number of taxonomic groups), or both within a sample^24^. Based on this analysis, no significant difference (*p* < 0.05) was detected in all the diversity indices (abundance-based coverage estimator (ACE), Chao1, Shannon, Simpson, Jackniff, and phylogenetic diversity) estimated in the high-altitude-adapted chicken lines, as shown in Fig. 6 and supplementary Table 2.

### Beta(β)-diversity

**Fig. 7:**
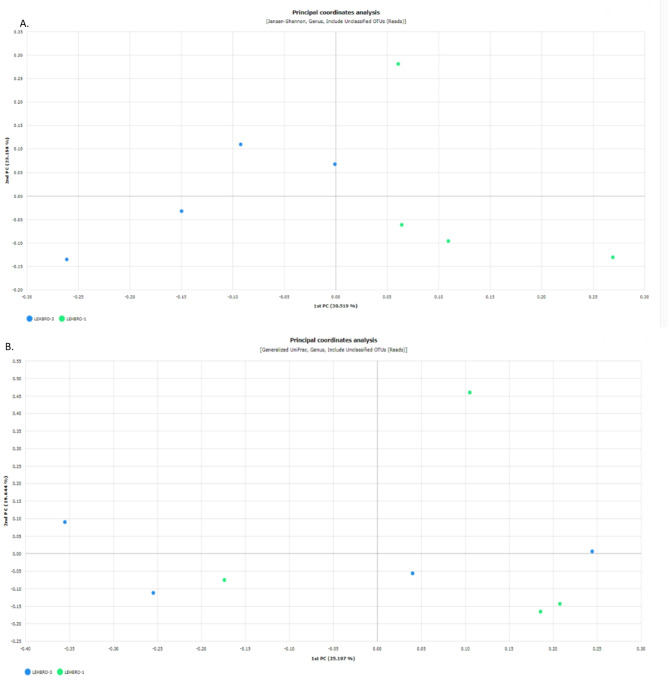
Beta (β)-diversity analysis of the gut bacteriome between two high-altitude-adapted chicken lines (at the genus level), using A. Jensen-Shannon and B. Generalized UniFrac algorithm.

To visualize the structural characteristics of the gut bacterial communities among the two different chicken lines, different algorithms, i.e., Jensen‒Shannon PCoA, Bray Curtis, UniFrac and generalized UniFrac PCoA, were studied as shown in Fig. 7 and supplementary Fig. 4.

**Fig. 8:**
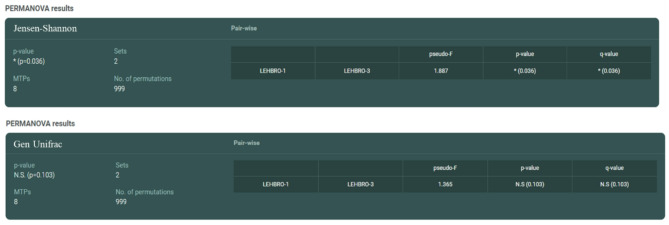
PERMANOVA-based comparison of beta diversity between the gut bacteriome of two high-altitude-adapted chicken lines (p-value < 0.05).

Thereafter, the significance of beta diversity was subsequently calculated via PERMANOVA (permutational multivariate analysis of variance) software to know the difference in microbial diversity between the two chicken lines, which indicated significant differences (*p* < 0.05) in beta diversity of gut microbes present in the high-altitude adapted lines. Consequently, the results revealed significant differences (*p* < 0.05) between the LEHBRO-1 and LEHBRO-3 chicken lines via the Jensen‒Shannon index (*p* = 0.036) at the genus level, however, no significant difference was observed in the generalized UniFrac (*p* = 0.103) as shown in Fig. 8.

### Functional profiling

In the present study, two databases were utilized to determine the functional profile of high-altitude-adapted chickens, i.e., PICRUSt, through the EzBioCloud server and the PICRUSt2 database. As per the EzBioCloud software, the functional pathways identified in the high-altitude-adapted chickens are mentioned in supplementary Tables 3 and 4. After statistical analysis, 28 pathways were identified through the Kruskal Wallis H Test, and 02 pathways through the Lefse test. Among these pathways, Amino sugar and Nucleotide metabolism, Glycolysis or gluconeogenesis, Starch and sucrose metabolism, and phosphotransferase system pathways were found to be upregulated in LEHBRO-3 than in LEHBRO-1. However, the results were not statistically different among the chicken lines after adjusting for multiple comparisons.

**Fig. 9:**
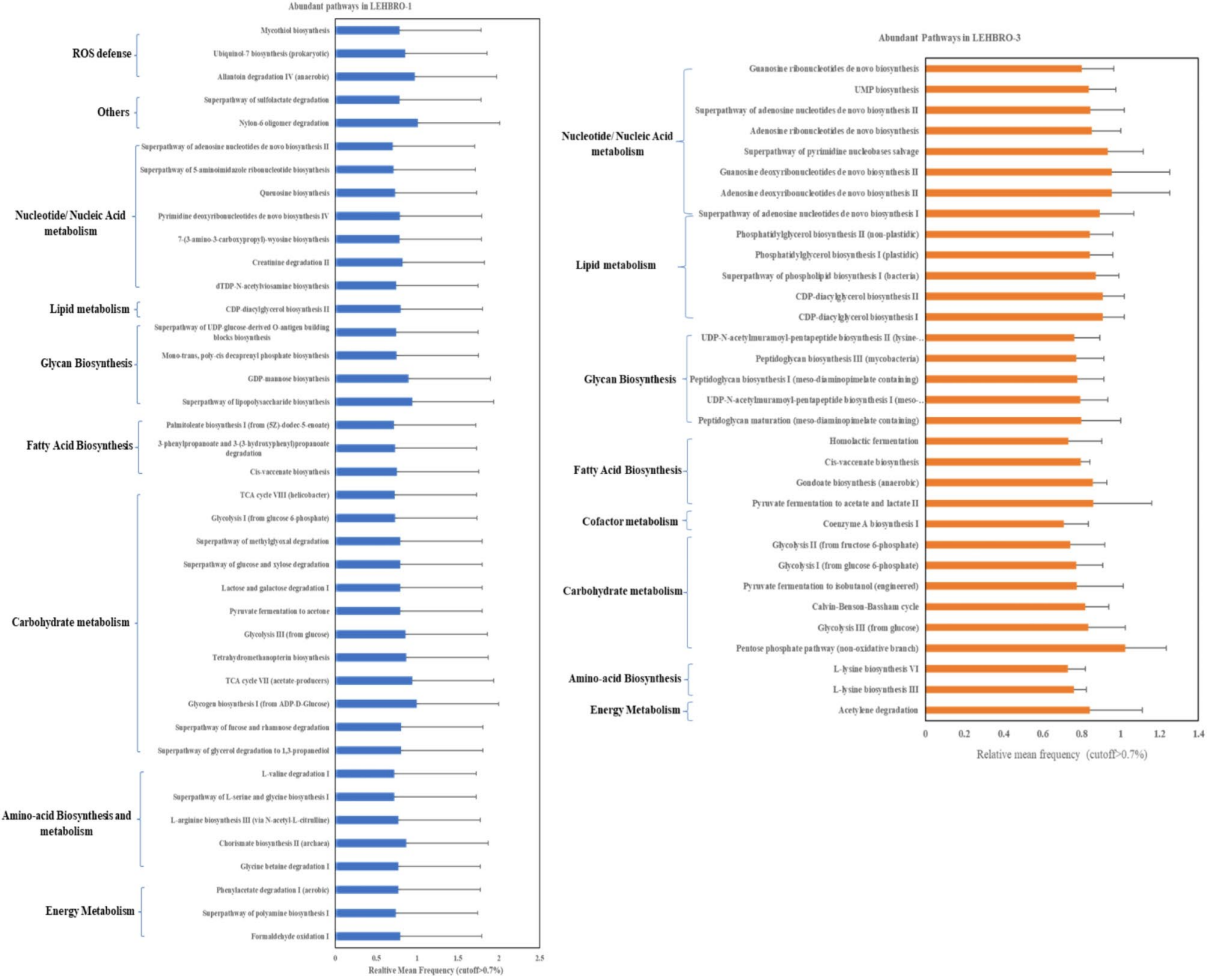
Graphical representation of metabolic pathways highly abundant in high-altitude-adapted chickens (cutoff > 0.7%). Note: *The ROS defense mechanism in LEHBRO-3 was less than 0.2% of the mean frequency.

In addition, the functional profile analysis of chicken gut bacteria through PICRUSt2 database revealed a total of 393 pathways, among which the pathways occurring with a relative abundance frequency of 0.7 cutoffs and above were considered to study the difference between the two adapted chicken lines, as shown in Fig. 9. In LEHBRO-1 chickens, a total of 370 pathways were found to be enriched, where 40 of which presented a relative abundance frequency of 0.7 cutoff and above. Among these pathways, 12 pathways were involved in carbohydrate metabolism, 07 in nucleotide metabolism, 05 in amino acid biosynthesis and metabolism, 04 in glycan biosynthesis, 03 in energy metabolism, 03 in the ROS defense mechanism, 03 in fatty acid biosynthesis, 01 in lipid biosynthesis, and 01 in other category pathways (Fig. 9). However, in LEHBRO-3 chickens, a total of 349 pathways were identified, 32 pathways of which occurred with a relative abundance of 0.7%. Among these pathways, 08 pathways were involved in nucleotide metabolism, 06 in carbohydrate metabolism, 05 in lipid biosynthesis, 05 in glycan biosynthesis, 04 in fatty acid biosynthesis, 02 in amino acid biosynthesis and metabolism, 01 in energy metabolism, and 01 in other categories.

**Fig. 10:**
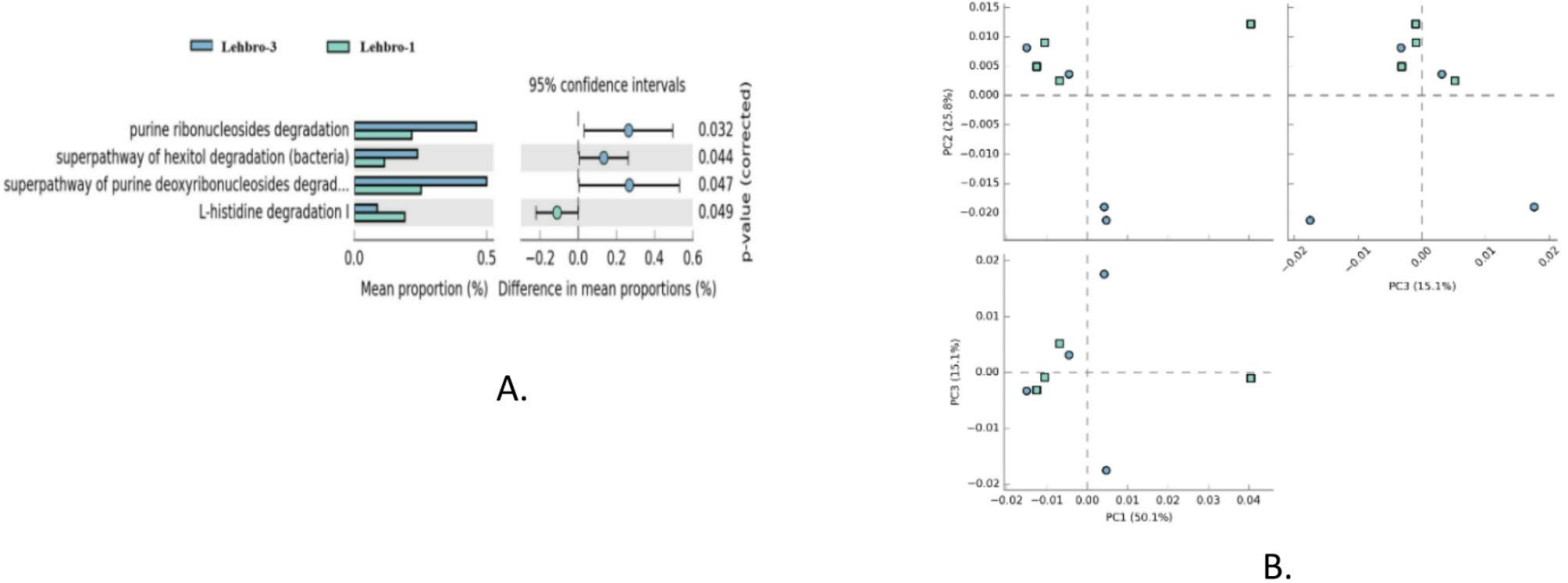
Differential analysis of metabolic pathways associated with the gut bacterial diversity of two chicken lines, i.e., LEHBRO-1 and LEHBRO-3, was conducted via statistical analysis of metagenomic profiles (STAMPs): (A) An extended error bar plot and (B) a PCA plot for two-group module comparison of PICRUSt-predicted KEGG function data.

Moreover, differences in the functional profiles of the gut bacteria between the chicken lines are shown in Fig. 10, which revealed significant differences in 04 pathways involving purine metabolism (purine ribonucleoside degradation and purine deoxyribonucleoside degradation) and protein metabolism (hexitol and L-histidine degradation).

Since the EzBioCloud database utilizes PICRUSt (2013)^25^ for functional analysis, it provides a lesser no. of pathways than the PICRUST2 database, which provides higher coverage of the pathways in the database with higher accuracy. Therefore, PICRUST2 results are discussed in the present study.

## Discussion

Researchers are increasingly interested in molecular, biochemical, and functional characteristics of high-altitude microbiotas due to their ability to thrive in harsh environmental conditions^26–28^. These microbiotas are known to have probiotic and metabolite-producing abilities that can enhance disease resistance, growth performance, and productivity of an animal^10,28^. While previous studies have highlighted differences between the gut microbial communities of high- and low-altitude animals, there remains a paucity of knowledge regarding the specific bacterial populations in different lines of high-altitude chickens, particularly those from Leh-Ladakh^1,28^. Therefore, this study aimed to bridge this knowledge gap by identifying potential gut microbiota in two high-altitude-adapted chicken lines through targeted amplicon sequencing and EzBioCloud-based 16 S MTP bioinformatics analysis. Additionally, this research also predicted the possible metabolic pathways present in the gut bacteriome in high-altitude-adapted chickens, providing insights into host-microbial interactions at high altitudes.

In the present study, microbiome taxonomic profiling across different taxon levels showed a notable prevalence of unculturable microbes in fecal samples from chickens, which substantiated the existence of a diverse array of microbial taxa that could not be successfully cultured via traditional methods. The presence of these unculturable microbes suggests the potential existence of novel gut bacterial species with unique functionalities, offering valuable insights into probiotic development and microbial biotechnology. In the present study, the taxonomic profile of bacterial communities at the phylum level revealed the significant predominance of phyla Firmicutes in the gut microbiome of high-altitude-adapted chickens, mirroring previous studies on high-altitude animals such as yaks, cattle, antelopes, pikas, sheep, chickens, dogs, and mice in the Tibetan region^8,10,11,29^. Conversely, the gut microbes of low-altitude animals are known to consist of a higher proportion of the phylum Bacteroidetes in their gut microbiome than the phylum Firmicutes^13,30,31^. In addition, similar to our results of Firmicutes-to-Bacteroidetes ratio, other studies have also shown that high-altitude animals have a significantly higher Firmicutes/Bacteroidetes (F/B) ratio, which is beneficial for energy harvesting in animals and can help the animal to maintain a metabolic balance and body homeostasis at high altitudes^32,33^. This finding also substantiates that the host regulates their metabolism through intestinal microbes to adapt to environmental changes^30^. Further, phyla proteobacteria were also found to be relatively abundant, followed by the phylum Bacteroidetes, and the phylum Actinobacteria in both chicken lines. The phylum Firmicutes, as evidenced in various studies, plays pivotal roles in maintaining gut health, host homeostasis, gut microbial balance, nutrient metabolism, essential nutrient production, such as that of vitamins, immune regulation, and protection against pathogens^34,35^. Hence, they have emerged as an essential probiotic candidate for promoting the overall health and body physiology of animal hosts. On the other hand, the phylum Proteobacteria is mostly found as one of the most abundant microbes in the guts of mammals and involves diverse groups with roles in nitrogen fixation, carbon cycling, and beneficial functions related to catabolizing feed into more nutrients and energy^36^, however, its abnormal expansion could lead to an imbalanced gut^37^. Furthermore, the bacterial flora of the phylum Bacteroidetes is known for degrading complex carbohydrates, aiding in digestion, and producing beneficial metabolites^38^. This study also observed the abundance of another phylum Actinobacteriota, in both lines of chickens, which is known to contribute to gut health, digestion, and protection against pathogens through the breaking of complex carbohydrates, producing antimicrobial compounds^39^. While Actinobacteriota may not be as abundant as other bacterial phyla, such as Firmicutes and Bacteroidetes, they still play important roles in gut health and metabolic functions^40,41^. In fact, the lower proportion of Actinobacteriota in high-altitude chickens might be because of increased altitude and hypoxia, which decrease the aerobic bacterial populations^32^. In the present study, the abundance of all these bacterial phyla in chickens was indicative of high-altitude adaptation. Further, an abundance of phyla Planctomycetes, and Parcubacteria OD1 was also observed in LEHBRO-3 chickens, where the proportion of Planctomycetes phylum is proportionately lesser in number than the low-altitude animals due to altitudinal effects^42,43^ and higher prevalence of Parcubacteria OD1 is because of their growing potential in a broad range of anoxic environments to help in energy and nutrient conservation, however, they lack any biosynthetic capabilities and may not have a direct role in the health and growth of the host^44^. Overall, these findings indicated that high-altitude chickens harbor a rich abundance of good microbes, i.e., Firmicutes, which could be harnessed to improve the adaptation and performance of low-altitude chickens at high altitudes. These findings showed a line-specific variation among the abundance of gut bacterial phyla, which substantiated that different gut microflora dominate in different lines of chickens reared at Leh Ladakh. These findings are compliant with those of previous studies, which stated differences in gut microbial diversity with differences in chicken breeds and lines^45,46^. Similarly, taxonomic analysis of gut bacterial communities at the genus level revealed that high-altitude chickens host a diverse range of genera in high-altitude adapted chicken lines. In addition, a huge difference in bacterial diversity was observed at the genus level between these two chicken lines. In addition, the findings of LEfSe analysis also disclosed and substantiated the line-specific differences in gut bacteriome of high-altitude-adapted chickens.

Among the identified genus of commensal gut microbes in high altitude adapted chickens, genus *Psychrobacter*, *Enterococcus*, *Carnobacterium*, *Lactobacillus*, *Clostridium*, and *Acinetobacter* are known for their beneficial probiotic and bioprotective properties to modulate immunity and production performance in animals^47–55^, genus *Brevibacterium*,* Pedobacter*,* Lactobacillus*,* Enterococcus*,* Aeriscardovia*,* Carnobacterium*,* Acinetobacter*,* Escherichia*, *Lysobacter*,* Pedobacter* and *Psychrobacter* are known for producing bioactive compounds^56–63^ and, genus *Mobilisporobacter* is known as a phosphate solubilizer^64^. In addition, the genus *Atopostipes* is known for reducing antibiotic resistance genes^65^, genus *Romboutsia* is known for its broad range of metabolic capabilities in carbohydrate utilization, fermentation, anaerobic respiration, and metabolic end products^66^ and genus *Turicibacter* is known for its anti-inflammatory and anti-tumorigenic properties in intestinal cancers^67^. Therefore, it could be stated that high-altitude adapted chickens contain a higher proportion of beneficial gut microbes that exert health-promoting benefits under high altitude conditions as these microbes have developed unique functional roles in immunity, metabolism, health, and adaptation of the host at high-altitude in comparison to the isolates from low-altitude. As a result, these commensal gut microbes could be exploited as next-generation probiotics for enhancing the adaptation and production performance of chickens at high altitudes.

Further, alpha and beta diversity are crucial metrics in microbial ecology that provide valuable insights into the structure, composition, and dynamics of microbial communities within ecosystems. Therefore, to assess the alpha diversity in the samples, different indices, such as the Shannon, Simpson, ACE, and Chao1 indices, were tested in the present study. The Shannon diversity index is known to measure the diversity of species, evenness, and richness in a community, whereas the ACE and Chao1 indices help measure species richness, and Simpson’s diversity index measures species diversity in a community^68^. Further, Phylogenetic diversity is a measure of biodiversity that incorporates phylogenetic differences between species, whereas the jackniff method provides a way to estimate diversity indices and their associated standard deviations^69^. Interestingly, no significant differences were detected between the samples of chicken lines, however, the specific types of microbe’s present (composition) in both chicken lines differed as reflected by the difference observed in the taxonomic composition. This could be because both breeds are kept under the same ecosystem i.e., environmental conditions, housing management, and diet, resulting in less variation in the richness and evenness of the microbial communities, but the types of species (or their proportions) may vary.

In addition, the beta diversity of bacterial communities between the two lines of chickens was compared using different algorithms, i.e., Generalized UniFrac, and Jensen–Shannon divergence. Since the generalized Unifrac (GUniFrac) measures dissimilarity based on the evolutionary relatedness of taxa, with additional parameters such as abundance weighting, and phylogenetic tree scaling, to provide more flexibility in capturing community differences by considering both the presence/absence of taxa and their relative abundances^70^. Comparatively, Jensen-Shannon divergence is a statistical measure that quantifies the difference between two probability distributions. Indeed, it is used for comparing microbial community profiles, it is not directly tied to taxonomic composition but focuses on the distribution of taxon abundances^71^. Therefore, the current findings emphasize the difference in taxonomic abundance, not microbial diversity, and substantiate the differences in the gut bacterial community structure between the two chicken lines adapted to high-altitude conditions.

Lastly, the present findings on the functional profile of the gut microbes of high-altitude chickens add another dimension to characterizing the differences in microbial communities in terms of their metabolic differences between the two lines of high-altitude chickens. The gut bacterial communities of both chicken lines were mostly enriched in pathways such as carbohydrate metabolism, nucleotide metabolism, lipid metabolism, fatty acid biosynthesis, amino acid biosynthesis and metabolism, energy metabolism, and glycan biosynthesis, which could be beneficial for meeting the high nutritional and metabolic requirements of the gut microbial community and host’s energy requirement at high altitudes^13,32,33,72^. The present study also revealed that the gut microbes of LEHBRO-1 chickens possessed more abundance of ROS defense mechanisms than those of LEHBRO-3 chickens, which might be because of differences in their egg-laying performance at high altitudes. Further, significant differences between the gut microbes of high-altitude-adapted chicken lines revealed significant upregulation of L-histidine degradation in LEHBRO-1, possibly because the activities of intestinal bacteria can convert amino acids, such as L-histidine, into biogenic amines, such as histamine, that suppress pro-inflammatory cytokine production by impeding MAP kinase signaling, an anti-oxidative mechanism useful for LEHBRO-1 chicken lines in the laying period as well as in high-altitude environments and must be responsible for better growth performance at high altitude^73^. Comparatively, in the LEHBRO-3 chicken lines, there was a significant upregulation of pathways, i.e., the degradation of purine ribonucleotides, purine de-ribonucleosides, and hexitol. These findings suggest that the upregulated hexitol degradation in the LEHBRO-3 chicken lines may be necessary to produce more intermediates for the glycolysis pathway^74^ to meet the energy requirements and enhance their growth performance at high altitudes. In addition, the increased purine degradation in these chicken lines may be associated with the presence of anaerobic purine-utilizing organisms, including Firmicutes and Proteobacteria, for evidently recycling purine nitrogen for purine homeostasis and host health^75,76^.

Overall, our study significantly advances the understanding of gut bacterial diversity in high-altitude-adapted chickens and their role in cellular metabolism and adaptation based on the identified pathways. This study also revealed that unique gut bacteriomes and their functions may be closely related to environmental host adaptation under high-altitude conditions, which could be exploited to improve the productivity of chickens. Further, the unique findings of this research will help in futuristic studies in these breeds with larger sample sizes to better understand the functionality of gut microbes in mechanisms of host organisms to survive and thrive there.

## Conclusions

The present findings provide new insights into the gut diversity composition in the high-altitude-adapted chicken lines (LEHBRO-1 and 3) and their potential functional cellular pathways present in these species. The taxonomic analysis revealed a significant abundance of Firmicutes in high-altitude chickens. The results of functional profiling of bacterial communities revealed enrichment in pathways related to carbohydrate, nucleotide, lipid, fatty acid, amino acid, and glycan metabolism, indicating the significant role of these microbes in supporting the resilience of adapted chickens to high-altitude conditions. These study findings would be helpful in developing novel probiotics and bacterial secretary bioactive compounds for boosting the health and productivity of chickens, ensuring sustainable poultry farming in challenging high-altitude environments. Furthermore, the diverse bacterial taxonomies of these two lines of chickens from those of low-altitude populations not only lay the foundation for future culture-dependent studies but also highlight the practical implications of our research on poultry production and the probiotics industry.

## Electronic supplementary material

Below is the link to the electronic supplementary material.

Supplementary Material 1

## Data Availability

The datasets generated and analyzed during the current study are available in the Sequence Read Archive bio-project of the National Centre for Biotechnology Information repository: PRJNA1163362.
