# Supplementary material for: Gut bacteriome dynamics in high altitude-adapted chicken lines: a key to future poultry therapeutics
Source: Sci Rep. 2025 Apr 7;15:11910. doi: 10.1038/s41598-025-96178-1 (PMC11976950; doi:10.1038/s41598-025-96178-1)
Supplement: Supplementary file 1 — Supplementary Material 1 [file 41598_2025_96178_MOESM1_ESM.docx]

**Supplementary Figure 1.** Comparative relative abundance of gut bacterial diversity at the Family level in high altitude adapted chicken lines (cutoff > 1%).

**Supplementary Figure 2.** Comparative relative abundance of gut bacterial diversity at the class level in high altitude adapted chicken lines (cutoff > 1%).

**Supplementary Figure 3.** Comparative relative abundance of gut bacterial diversity at the order level in high altitude adapted chicken lines (cutoff > 1%).

**Supplementary Figure 4.** Bray Curtis and Unifrac analysis for Beta Diversity in High-altitude- adapted Chickens.

**Supplementary Table 1.** Total reads, average length, and operational taxonomic units (OTUs) among different samples collected from the high-altitude-adapted DIHAR chicken lines.

**Supplementary Table 2.** Alpha diversity in high-altitude-adapted chicken lines

**Supplementary Table 3.** Ezbiocloud-based Functional profile analysis between gut bacteriome of two high-altitude adapted chicken lines using Kruskal Wallis H test.

**Supplementary Table 4.** Ezbiocloud-based Functional profile analysis between gut bacteriome of two high-altitude adapted chicken lines using Lefse.


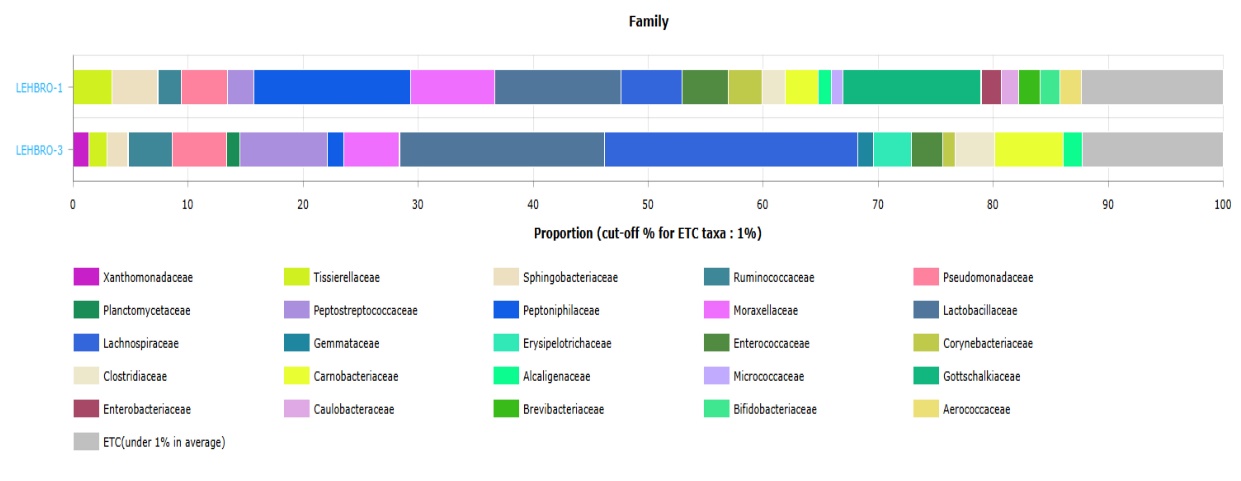
**Supplementary Figure 1.** Comparative relative abundance of gut bacterial diversity at the Family level in high altitude adapted chicken lines (cutoff > 1%).


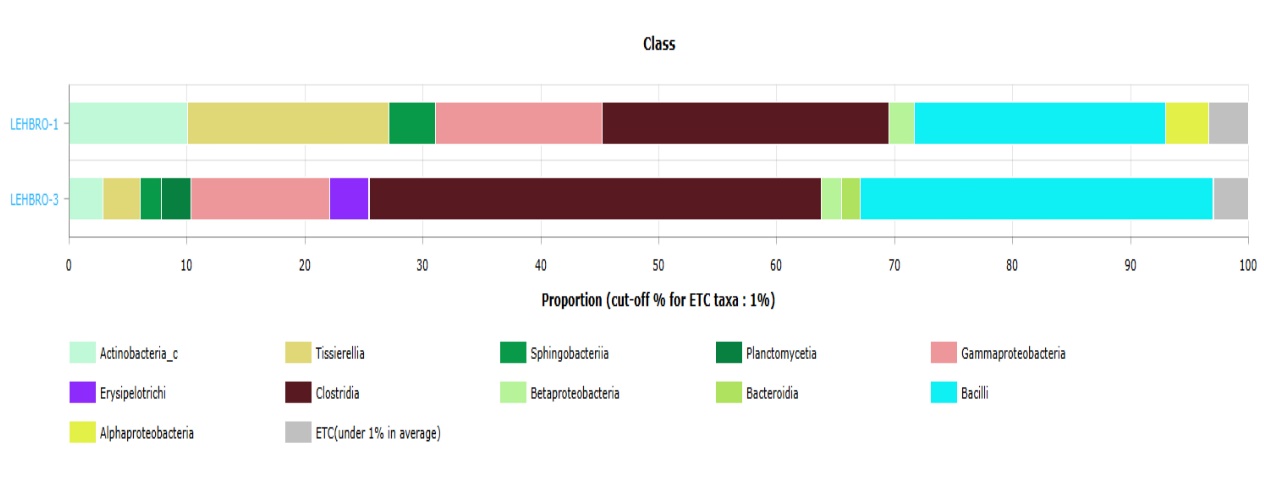
**Supplementary Figure 2.** Comparative relative abundance of gut bacterial diversity at the class level in high altitude adapted chicken lines (cutoff > 1%).

**Supplementary Figure 3.** Comparative relative abundance of gut bacterial diversity at the order level in high altitude adapted chicken lines (cutoff > 1%).


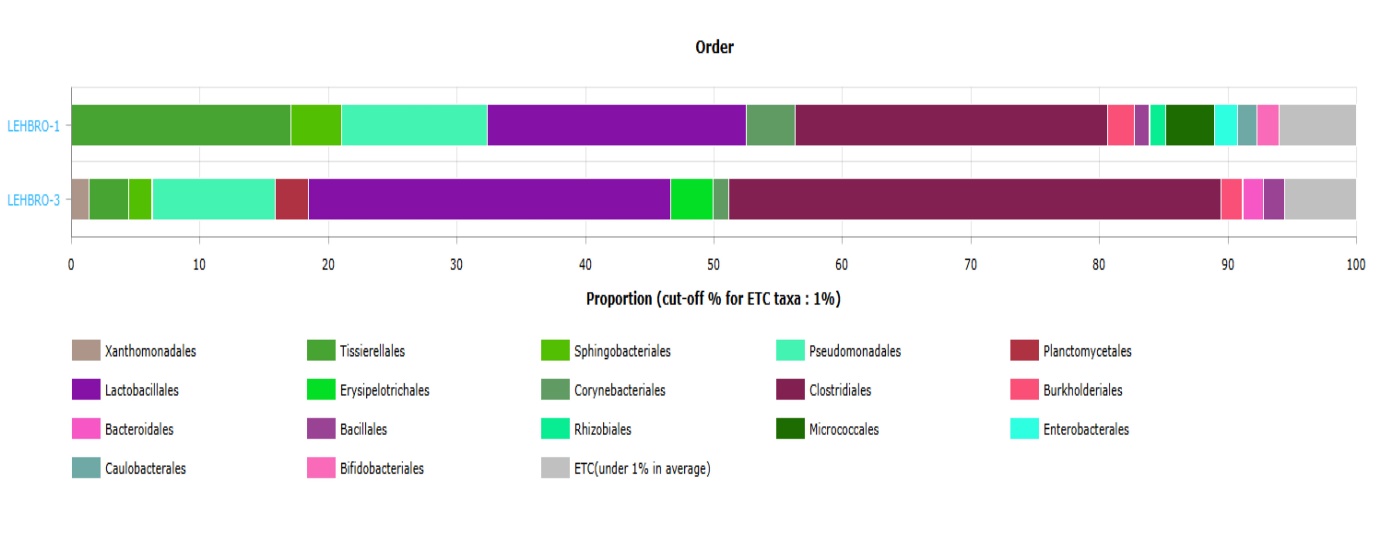


**Supplementary Figure 4.** Bray Curtis and Unifrac analysis for Beta Diversity in High altitude- adapted Chickens.

**
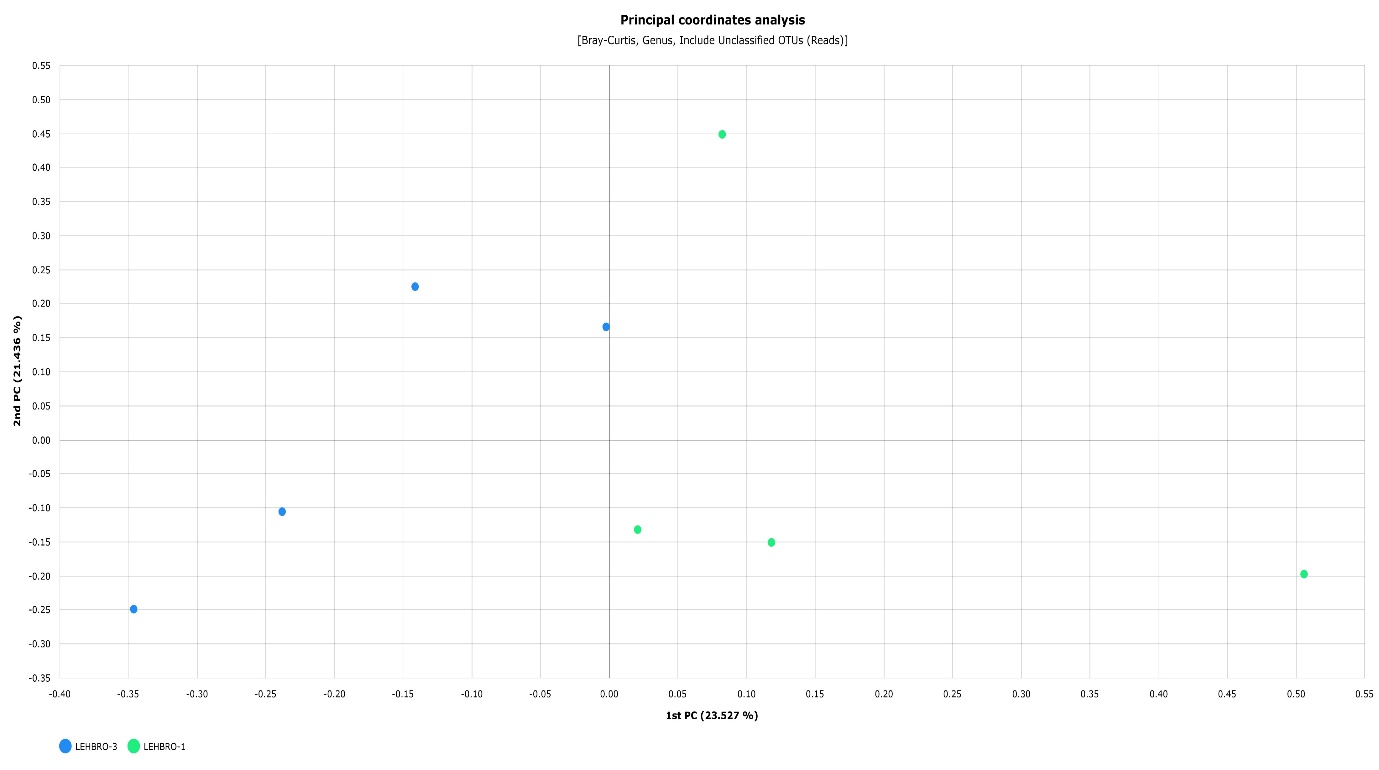
**

**
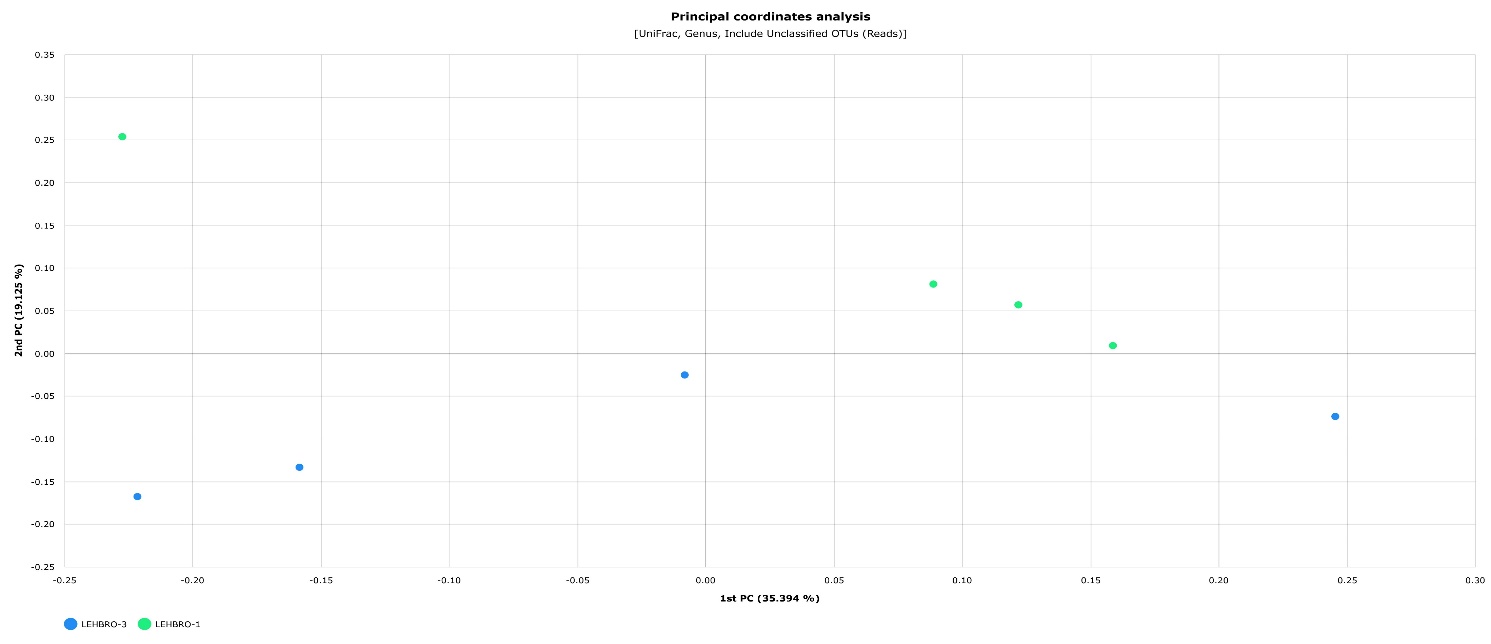
**

**Supplementary Table 1.** Total reads, average length, and operational taxonomic units (OTUs) among different samples collected from the high-altitude-adapted DIHAR chicken lines.

| **S. No.** | **Sample name** | **Reads (Q>20)** | **Total valid reads** | **Average read length** | **No. of OTUs found @97% cutoff** |
| --- | --- | --- | --- | --- | --- |
| 1. | LEHBRO-1 (1) | 69584 | 41231 | 409.7bp | 3108 |
| 2. | LEHBRO-1 (2) | 100000 | 75068 | 419bp | 4082 |
| 3. | LEHBRO-1 (3) | 100000 | 71896 | 425.9bp | 3848 |
| 4. | LEHBRO-1 (4) | 100000 | 74133 | 425.5bp | 3994 |
| 5. | LEHBRO-3 (1) | 100000 | 83102 | 422.7bp | 3742 |
| 6. | LEHBRO-3 (2) | 100000 | 75486 | 428.6bp | 4505 |
| 7. | LEHBRO-3 (3) | 100000 | 80462 | 416.3bp | 3221 |
| 8. | LEHBRO-3 (4) | 81960 | 65298 | 424.5bp | 3896 |

**Supplementary Table 2.** Alpha diversity in high-altitude adapted chicken lines

| **S. No.** | **Sample** | **ACE** | **Chao1** | **Jackknife** | **Shannon** | **Simpon** | **No. of OTUs** | **Phylogenetic Diversity** | **Good Coverage (%)** |
| --- | --- | --- | --- | --- | --- | --- | --- | --- | --- |
| 1. | LEHBRO-1_1 | 3156.8 | 3114.5 | 3233.0 | 5.154 | 0.121 | 3108 | 790.0 | 99.7 |
| 2. | LEHBRO-1_2 | 4103.0 | 4083.1 | 4143.0 | 5.957 | 0.016 | 4082 | 961.0 | 99.9 |
| 3. | LEHBRO-1_3 | 3902.4 | 3854.5 | 3993.0 | 5.341 | 0.021 | 3848 | 1020.0 | 99.8 |
| 4. | LEHBRO-1_4 | 4035.9 | 3998.1 | 4105.0 | 5.826 | 0.015 | 3994 | 744.0 | 99.9 |
|  | **Average** | **3799.53** | **3762.55** | **3868.5** | **5.569** | **0.043** | **3758** | **878.75** | **99.83** |
| 5. | LEHBRO-3_1 | 3769.2 | 3743.9 | 3816.0 | 4.883 | 0.048 | 3742 | 890.0 | 99.9 |
| 6. | LEHBRO-3_2 | 4530.4 | 4506.4 | 4576.0 | 5.945 | 0.016 | 4505 | 977.0 | 99.9 |
| 7. | LEHBRO-3_3 | 3261.9 | 3225.6 | 3333.0 | 3.941 | 0.088 | 3221 | 702.0 | 99.9 |
| 8. | LEHBRO-3_4 | 3953.2 | 3903.4 | 4049.0 | 5.118 | 0.039 | 3896 | 1235.0 | 99.8 |
|  | **Average** | **3878.68** | **3844.83** | **3943.5** | **4.972** | **0.048** | **3841** | **951.0** | **99.88** |

**Supplementary Table 3.** Ezbiocloud-based Functional profile analysis between gut bacteriome of two high-altitude adapted chicken lines using Kruskal Wallis H test

| **S. No.** | **Pathway (Kruskal walis-H test )** | **Definition** | **Module** | **Orthology** | **p-value** | **p-value (FDR)** | **LEHBRO-1** | **LEHBRO-3** |
| --- | --- | --- | --- | --- | --- | --- | --- | --- |
|  | ko00520 | Amino sugar and nucleotide sugar metabolism | M00361, M00362, M00265, M00266, M00267, M00282, M00549, M00761, M00554, M00809 | K00884, K00972, K01809, K02377, K02777, K02794, K02795, K02796, K07106, K10012, K12453, K15779, K18429, K18430, K18431, K20116, K20117, K20118 | 0.043 | 0.688 | 0.807 | 1.039 |
|  | ko00010 | Glycolysis / Gluconeogenesis | M00265, M00266, M00272, M00002, M00003, M00001, M00114, M00307, M00809 | K00149, K00150, K01223, K02777, K03737, K04022, K13952, K15635, K15779, K16370, K20116, K20117, K20118 | 0.043 | 0.688 | 0.752 | 0.910 |
|  | ko00500 | Starch and sucrose metabolism | M00266, M00269, M00270, M00275, M00565, M00855, M00806, M00854 | K01194, K01200, K01208, K01223, K01232, K02760, K02761, K02777, K02809, K02810, K02818, K02819, K05992, K15779, K16055, K20812, K22451 | 0.043 | 0.688 | 0.624 | 0.913 |
|  | ko00220 | Arginine biosynthesis | M00028, M00029, M00763, M00844, M00845 | K00145, K00611, K00620, K00821, K05597, K05831, K13240, K14048, K14454 | 0.043 | 0.688 | 0.410 | 0.356 |
|  | ko00770 | Pantothenate and CoA biosynthesis | M00119, M00120 | K00207, K00606, K00826, K01464, K01652, K01653, K01918, K01947 | 0.021 | 0.666 | 0.386 | 0.324 |
|  | ko02060 | Phosphotransferase system (PTS) | M00304, M00305, M00265, M00266, M00267, M00268, M00269, M00270, M00271, M00272, M00273, M00274, M00275, M00279, M00277, M00278, M00280, M00281, M00282, M00283, M00287, M00764, M00303, M00610, M00276, M00807, M00809, M00806 | K02756, K02757, K02760, K02761, K02769, K02777, K02794, K02795, K02796, K02809, K02810, K02818, K02819, K20116, K20117, K20118 | 0.021 | 0.666 | 0.385 | 0.703 |
|  | ko04122 | Sulfur relay system |  | K03148, K03154, K03637 | 0.043 | 0.688 | 0.268 | 0.215 |
|  | ko00290 | Valine, leucine and isoleucine biosynthesis | M00019, M00535, M00570, M00432 | K00826, K01649, K01652, K01653, K01704, K09011 | 0.021 | 0.666 | 0.240 | 0.169 |
|  | ko00600 | Sphingolipid metabolism | M00066, M00099, M00100, M00067, M00094 | K01117, K01186, K01189, K01201, K01202, K04715 | 0.021 | 0.666 | 0.180 | 0.292 |
|  | ko04142 | Lysosome |  | K01136, K01186, K01189, K01192, K01201, K01202, K01373, K01565, K12307, K21398 | 0.043 | 0.688 | 0.117 | 0.147 |
|  | ko03013 | RNA transport | M00399, M00406, M00405, M00427, M00426, M00428, M00430 | K03249, K12875, K14314 | 0.043 | 0.688 | 0.106 | 0.120 |
|  | ko00960 | Tropane, piperidine and pyridine alkaloid biosynthesis |  | K00817, K01582, K14454 | 0.043 | 0.688 | 0.087 | 0.063 |
|  | ko00603 | Glycosphingolipid biosynthesis - globo and isoglobo series | M00068 | K01189 | 0.021 | 0.666 | 0.077 | 0.121 |
|  | ko04724 | Glutamatergic synapse |  | K05612, K05613, K05614, K07825, K08043 | 0.021 | 0.666 | 0.074 | 0.084 |
|  | ko04727 | GABAergic synapse |  | K05185, K07825, K08043, K15374 | 0.043 | 0.688 | 0.065 | 0.083 |
|  | ko00531 | Glycosaminoglycan degradation | M00076, M00077, M00078, M00079 | K01136, K01565 | 0.021 | 0.666 | 0.056 | 0.088 |
|  | ko00984 | Steroid degradation |  | K01822, K16046, K16049 | 0.021 | 0.666 | 0.052 | 0.031 |
|  | ko00572 | Arabinogalactan biosynthesis - Mycobacterium |  | K13686 | 0.021 | 0.666 | 0.041 | 0.025 |
|  | ko04612 | Antigen processing and presentation |  | K04079, K08056, K08065 | 0.043 | 0.688 | 0.040 | 0.020 |
|  | ko04940 | Type I diabetes mellitus |  |  | 0.021 | 0.666 | 0.036 | 0.029 |
|  | ko04011 | MAPK signaling pathway - yeast | M00516, M00687 | K17971, K19816 | 0.043 | 0.688 | 0.034 | 0.045 |
|  | ko04713 | Circadian entrainment |  | K07825, K08043, K13240 | 0.021 | 0.666 | 0.011 | 0.023 |
|  | ko05032 | Morphine addiction |  | K05185, K07825, K08043 | 0.021 | 0.666 | 0.008 | 0.021 |
|  | ko00073 | Cutin, suberine and wax biosynthesis |  | K17991 | 0.043 | 0.688 | 0.003 | 0.010 |
|  | ko04392 | Hippo signaling pathway - multiple species |  |  | 0.021 | 0.666 | 0.003 | 0.006 |
|  | ko00943 | Isoflavonoid biosynthesis |  | K05281 | 0.021 | 0.666 | 0.002 | 0.006 |
|  | ko04136 | Autophagy - other |  | K08269 | 0.043 | 0.688 | 0.001 | 0.001 |
|  | ko00402 | Benzoxazinoid biosynthesis |  | K13222 | 0.043 | 0.688 | 0.000 | 0.000 |

**Supplementary Table 4.** Ezbiocloud-based Functional profile analysis between gut bacteriome of two high-altitude adapted chicken lines using Lefse.

| **S. No.** | **Pathway (Lefse)** | **Definition** | **Module** | **Orthology** | **LDA effect size** | **p-value** | **p-value (FDR)** | **LEHBRO-1** | **LEHBRO-3** |
| --- | --- | --- | --- | --- | --- | --- | --- | --- | --- |
|  | ko00500 | Starch and sucrose metabolism | M00266, M00269, M00270, M00275, M00565, M00855, M00806, M00854 | K01223, K01232, K02760, K02761, K02809, K02810 | 3.141 | 0.043 | 0.688 | 0.624 | 0.913 |
|  | ko02060 | Phosphotransferase system (PTS) | M00304, M00305, M00265, M00266, M00267, M00268, M00269, M00270, M00271, M00272, M00273, M00274, M00275, M00279, M00277, M00278, M00280, M00281, M00282, M00283, M00287, M00764, M00303, M00610, M00276, M00807, M00809, M00806 | K02756, K02757, K02760, K02761, K02794, K02795, K02796, K02809, K02810 | 3.121 | 0.021 | 0.666 | 0.385 | 0.703 |
